# The sero-prevalence and virulence determinants of *Klebsiella pneumoniae* isolated from urine in outpatient units: a 20-year collection from Taiwan as a herald for serotype vaccine selection

**DOI:** 10.1128/spectrum.02633-25

**Published:** 2025-12-11

**Authors:** Sheng-Kang Chiu, Esther Yip-Mei Liu, Fu-Mei Lin, Jia-Je Li, Yu-Kuo Tsai, Chu-Hsuan Cheng, Ching Hsun Wang, Feng-Yee Chang, Jung-Chung Lin, L. Kristopher Siu, Hospitals’ Group for Taiwan Surveillance of Antimicrobial Resistance (TSAR)

**Affiliations:** 1 Division of Infectious Diseases, Department of Medicine, Taipei Tzu Chi Hospital, Buddhist Tzu Chi Medical Foundation, New Taipei City, Taiwan; 2 School of Medicine, Tzu Chi University 59216, Hualien, Taiwan; 3 Division of Infectious Diseases and Tropical Medicine, Department of Medicine, Tri-Service General Hospital, National Defense Medical University 71548 , Taipei, Taiwan; 4 Institute of Infectious Diseases and Vaccinology, National Health Research Institutes 50115, Miaoli, Taiwan; 5 Department of Nursing, Taipei Tzu Chi Hospital, Buddhist Tzu Chi Medical Foundation, New Taipei City, Taiwan; 6 Graduate Institute of Basic Medical Science, China Medical University, Taichung, Taiwan; Taichung Veterans General Hospital, Taichung, Taiwan

**Keywords:** *Klebsiella pneumoniae*, urinary tract infection, capsular polysaccharide serotypes, virulence determinants

## Abstract

This study analyzed 570 non-duplicate *Klebsiella pneumoniae* isolates (*n* = 570) from outpatient urine cultures collected between 1998 and 2018 through the Taiwan Surveillance of Antimicrobial Resistance (TSAR) program. Serotyping, antimicrobial susceptibility testing, and virulence gene profiling were performed. Results were compared with 521 previously reported bloodstream isolates collected in 1998, 2008, and 2018. The most common urinary tract infection (UTI) serotypes were K2, K64, K62, K1, and K25, with non-typeable isolates comprising 19.3%. Compared to bloodstream isolates, non-typeable strains were more frequent in UTIs, while K1 and K2 were more common in bloodstream infections. Resistance to cephalosporins, carbapenems, and ciprofloxacin increased significantly after 2014. K64 showed over 72.2% resistance to cephalosporins and rising quinolone resistance. In contrast, K1 and K2 exhibited low resistance but carried the highest burden of virulence genes. Non-typeable isolates lacked virulence determinants, suggesting lower pathogenic potential. This 20-year analysis highlights the distinct serotype and resistance patterns in community-onset UTI isolates in Taiwan and underscores the importance of incorporating prevalent, drug-resistant serotypes into future vaccine strategies.

**IMPORTANCE:**

*Klebsiella pneumoniae* is an important cause of urinary tract infections in the community. With rising resistance to commonly used antibiotics, preventive strategies, such as vaccination, are urgently needed. This 20-year analysis provides detailed data on the distribution of bacterial types and their resistance patterns in Taiwan. The findings offer valuable insight for selecting vaccine targets that cover the most prevalent and drug-resistant strains, supporting the development of effective preventive tools against multidrug-resistant infections.

## INTRODUCTION

*Klebsiella pneumoniae*, a Gram-negative bacterium, has gained significant attention because it causes a wide range of infections (1). Among community-acquired infections, pneumonia, liver abscess, and urinary tract infections (UTIs) are frequently reported (2, 3). Among these, *K. pneumoniae* is recognized as the second most common causative agent of community-onset UTIs, particularly affecting older adults and individuals with underlying conditions (4). These infections impose a considerable global health burden, sometimes leading to significant morbidity and mortality. Additionally, patients commonly face the challenges of recurrent infections or reinfection (5).

In light of growing antimicrobial resistance and recurrent infections, vaccine-based prevention strategies have garnered increasing interest. Capsular polysaccharide (CPS)-based vaccines targeting *K. pneumoniae* have shown promise in preclinical studies, with both traditional CPS formulations and novel bioconjugate platforms demonstrating immunogenicity and protective efficacy in animal models (6, 7). However, the diversity of capsular serotypes—over 80 identified to date—remains a major challenge. Rational serotype selection based on epidemiological prevalence is therefore critical to the success of vaccine development.

Understanding the seroepidemiology of *K. pneumoniae* in urinary tract infections is a key step toward effective vaccine formulation. Our previous study characterized the serotype distribution of bloodstream isolates in Taiwan (8). In this study, we extend our investigation to community-onset UTI isolates and explore whether significant differences exist between urine and blood culture serotypes. This 20-year surveillance analysis provides new insights into serotype dynamics, resistance patterns, and their potential implications for vaccine design and infection control strategies.

## RESULTS

### Serotyping of *K. pneumoniae* urine culture isolates in outpatient unit from 1998 to 2018, and comparison of prevalence to previously published blood culture isolates in the same period of time

Serotyping of the urine culture *K. pneumoniae* from outpatient department showed that the top 11 prevalent types were as follows: serotypes K2 (7.5%), K64 (6.8%), K62 (6.7%), K1 (4.6%), K25 (4.0%), K20, K28, and K54 (3.9% of each), K24 (3.5%), K57, and K15 (2.8% of each) which constituted 41.6% in total isolates (Table 1). Additionally, a significant proportion (19.3%) of isolates was classified as non-typeable in this study. Apart from the top 10 prevalent serotypes, other serotypeable isolates constituted ≤2.6% for each individual type. Throughout the 20 years of collection, serotypes K4, K6, K11, K29, K32, K36, K37, K40, K41, K44, K49, K50, K56, K59, K65-72, K79, K81, and K82 were not identified in any year of survey, indicating their rarity in community-onset UTI.

**TABLE 1:** Serotyping of UTI *K. pneumoniae* collected from 1998 to 2018^*a*^

Serotype	No. of isolates in the year			Total (%)
	1998–2004 (*n* = 131)	2006–2012 (*n* = 225)	2014–2018 (*n* = 214)	
**K1**	**5**	**9**	**12**	**26** (**4.6**)
**K2**	**15**	**17**	**11**	**43** (**7.5**)
K3	2	4	0	6 (1.1)
K5	1	4	3	8 (1.4)
K7	1	4	1	6 (1.1)
K8	2	1	1	4 (0.7)
K9	1	3	1	5 (0.9)
K10	2	1	0	3 (0.5)
K12	2	2	0	4 (0.7)
K13	0	1	2	3 (0.5)
K14	1	2	1	4 (0.7)
**K15**	**5**	**7**	**4**	**16** (**2.8**)
K16	6	1	8	15 (2.6)
K17	2	2	2	6 (1.1)
K18	4	1	0	5 (0.9)
K19	0	1	0	1 (0.2)
**K20**	**8**	**8**	**6**	**22** (**3.9**)
K21	1	1	0	2 (0.4)
K22	0	3	0	3 (0.5)
K23	1	1	3	5 (0.9)
**K24**	**3**	**8**	**9**	**20** (**3.5**)
**K25**	**11**	**8**	**4**	**23** (**4.0**)
K26	2	0	0	2 (0.4)
K27	1	1	4	6 (1.1)
**K28**	**7**	**6**	**9**	**22** (**3.9**)
K30	0	3	2	5 (0.9)
K31	1	2	0	3 (0.5)
K33	0	0	1	1 (0.2)
K34	1	1	0	2 (0.4)
K35	0	1	6	7 (1.2)
K38	0	2	5	7 (1.2)
K39	0	3	3	6 (1.1)
K42	0	2	0	2 (0.4)
K43	0	3	2	5 (0.9)
K45	0	1	1	2 (0.4)
K46	0	0	1	1 (0.2)
K47	0	0	9	9 (1.6)
K48	0	1	0	1 (0.2)
K51	0	1	3	4 (0.7)
K52	0	1	1	2 (0.4)
K53	0	1	1	2 (0.4)
**K54**	**9**	**7**	**6**	**22** (**3.9**)
K55	0	0	1	1 (0.2)
**K57**	**4**	**3**	**9**	**16** (**2.8**)
K58	0	1	0	1 (0.2)
K60	1	0	0	1 (0.2)
K61	1	1	1	3 (0.5)
**K62**	**8**	**19**	**11**	**38** (**6.7**)
K63	2	1	4	7 (1.2)
**K64**	**3**	**18**	**18**	**39** (**6.8**)
K74	0	1	1	2 (0.4)
K80	0	1	2	3 (0.5)
KN2	1	7	0	8 (1.4)
Non-typeable	17	48	45	110 (19.3)


^*a*^ Bold, 11 most common serotypes in two decades of *K. pneumoniae* urine cultures.

### Comparison between the blood culture collection and urine culture collection from the same period of time

**Fig 1:**
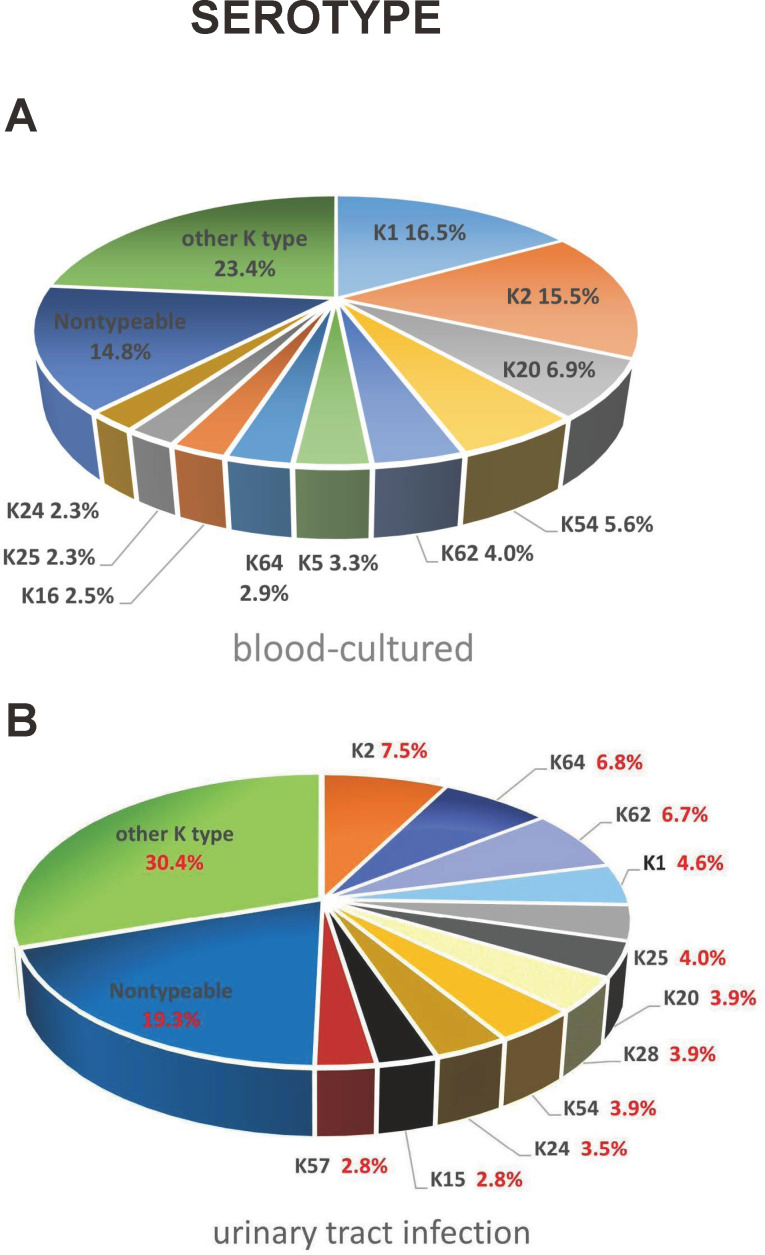
The distribution of serotypes between the previously published isolates from blood culture (**A**) and isolates in the present study from UTI (**B**).

When comparing to the previously published blood culture data (8), there were several significant differences in the distribution of serotypes and frequency of occurrence between the blood and urine cultured isolates (Fig. 1). Non-typeable isolates frequently appeared in both UTI and blood culture; 19.3% was identified in UTI and 14.8% was found in blood culture isolates (Fig. 1A and B). In UTI, non-typeable isolates were the most frequently identified types and more prevalent than the invasive serotype K1 and K2 isolates (19.3% vs 4.6% and 7.5%). On the contrary, non-typeable isolates were less frequently identified than the invasive serotype K1 and K2 (14.8% vs 16.5% and 15.5%) in blood culture isolates. In comparison of sero-prevalence between UTI and blood culture isolates in the same period of time, serotype K2 followed by K64, K62, K1, K25, K20, K28, and K54, K24, K57, and K15 were the top 10 frequently isolated serotypes in UTI while the order of serotype in blood culture isolates was K1 followed by K2, K20, K54, K62, K5, K64, K16, K24, and K25 were the top 10 frequently isolated serotypes in blood culture (Fig. 1A and B). Serotypes K1, K2, K20, K24, K54, K62, and K64 were listed within the top 10 prevalent serotypes in both UTI and blood culture, but the proportion of isolates in total and distribution of order were different between blood culture and UTI (Table 2). In accordance with the top ten prevalent serotypes in either blood culture or UTI, the 13 serotypes covered at least 54.4% of infection in both bacteremia and UTI (Table 2). By using Chi’s square analysis to isolate from the top 10 prevalence in either blood culture or urine culture, serotypes K1, K2, and K20 were significantly more prevalent (*P* < 0.05) in blood culture than in urine culture, while K28 and K64 were significantly (*P* < 0.05) more prevalent in urine culture than blood culture (Table 2), indicating the infectivity was different between bacteremia and urinary tract infection.

**TABLE 2:** Comparison of differences in the serotype between isolates from blood cultured from a previous study (8) and urine cultured isolates in this study

Serotype^*a*^	Blood (*n* = 521)	UTI (*n* = 570)	*P* value*^b^*
K1	86 (16.5%)	26 (4.6%)	**<0.001^*b*^**
K2	81 (15.5%)	43 (7.5%)	**<0.001^*b*^**
K5	17 (3.3%)	8 (1.4%)	0.064
K15	10 (1.9%)	16 (2.8%)	0.446
K16	13 (2.5%)	15 (2.6%)	1
K20	36 (6.9%)	22 (3.9%)	**0.035^*b*^**
K24	12 (2.3%)	20 (3.5%)	0.317
K25	12 (2.3%)	23 (4.0%)	0.147
K28	4 (0.8%)	22 (3.9%)	**0.001^*b*^**
K54	29 (5.6%)	22 (3.9%)	0.234
K57	8 (1.5%)	16 (2.8%)	0.221
K62	21 (4.0%)	38 (6.7%)	0.073
K64	15 (2.9%)	39 (6.8%)	**0.004^*b*^**
Total	344 (66.0%)	310 (54.4%)	
Other K types^*c*^	100 (19.2%)	150 (26.3%)	**<0.001^*b*^**
Non-typable	77 (14.8%)	110 (19.3%)	0.057

^*a*^ The serotypes with the top 11 prevalence, either from blood or urine cultures, were listed individually to compare statistically significant differences. Other K types or non-typable strains were grouped together for comparison.

^*b*^ *P* value <0.05 represents a statistically significant difference and is in bold.

^*c*^ K-antigen serotypes are not included among those listed in the table.

### The trend of susceptibility change of UTI isolates over the two decades

**Fig 2:**
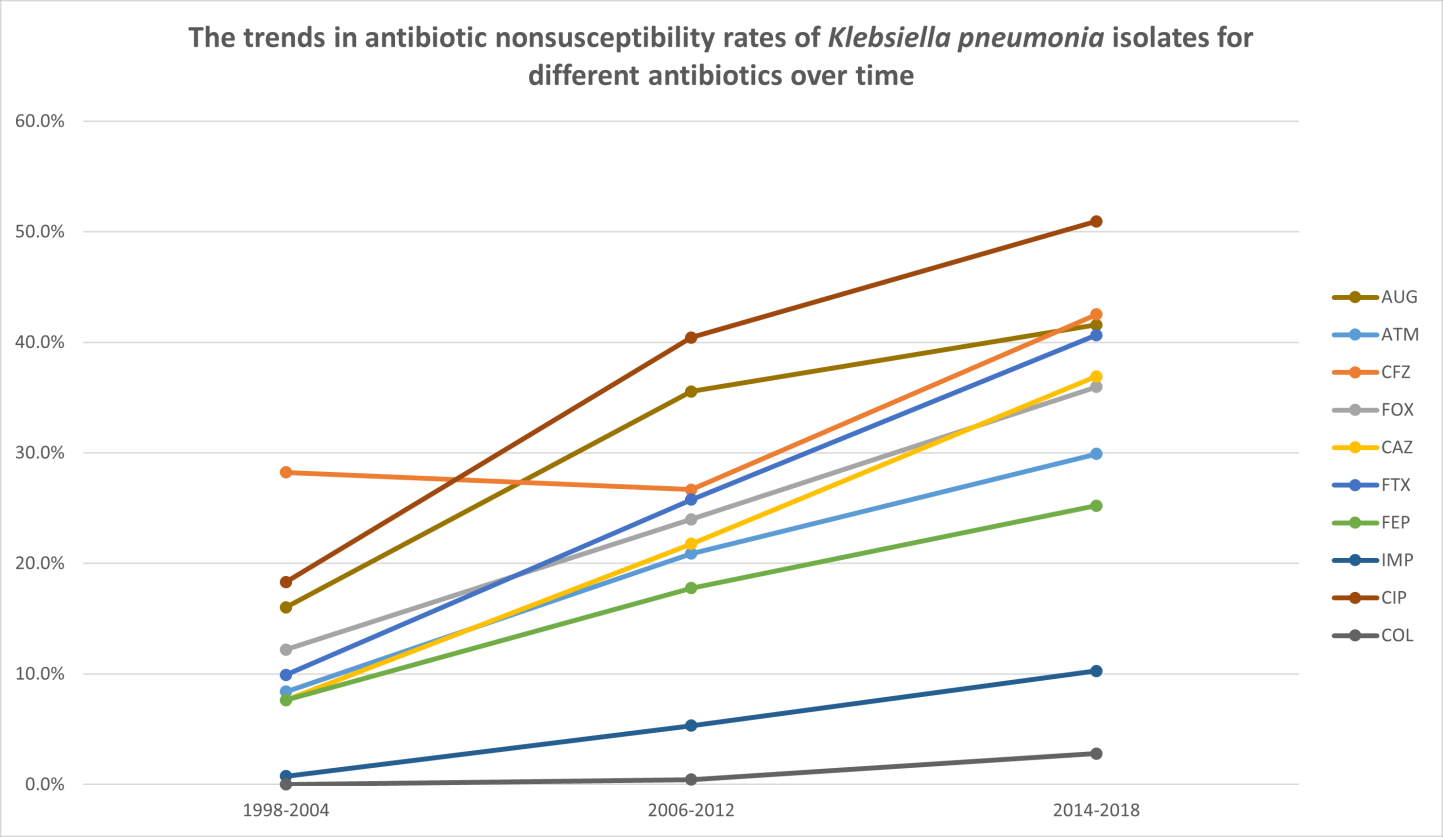
The antimicrobial susceptibility trend of UTI isolates collected from outpatient units. Only antibiotics showing statistically significant changes in non-susceptibility rates over time are presented. AUG, amoxicillin-clavulanate; ATM, aztreonam; CFZ, cefazolin; FOX, cefoxitin; CAZ, ceftazidime; CTX, cefotaxime; FEP, cefepime; IMP, imipenem; CIP, ciprofloxacin; COL, colistin.

Over the 20-year surveillance period (1998–2018), *K. pneumoniae* isolates from outpatient urinary tract infections exhibited a significant increase in resistance to multiple antibiotics (Table 3 and Fig. 2). Notably, resistance to aztreonam (ATM) surged from 8.4% to 29.9% (*P* < 0.001), while cefazolin (CFZ) resistance increased from 27.5% to 47.7% (*P* < 0.001). Similar upward trends were observed for cefoxitin (FOX, 13.7% to 36.0%), ceftazidime (CAZ, 9.2% to 36.9%), cefotaxime (FTX, 9.9% to 40.7%), and cefepime (FEP, 6.9% to 24.8%), all showing statistically significant increases (*P* < 0.001). Imipenem (IMP) resistance rose from 0.8% to 10.3%, signaling growing carbapenem resistance. Ciprofloxacin (CIP) resistance more than doubled, rising from 19.1% to 50.9% (*P* < 0.001), and resistance to amoxicillin-clavulanate (AUG) nearly doubled, from 19.8% to 41.6% (*P* < 0.001).

**TABLE 3:** The 20 years susceptibility trend of UTI isolates collected from the outpatients’ unit

Antibiotic^*a*^	Year, non-susceptibility rate (%)			*P* value*^b^*
	1998–2004	2006–2012	2014–2018	
AUG	16.0	35.6	41.6	**<0.001^*b*^**
ATM	8.4	20.9	29.9	**<0.001^*b*^**
CFZ	28.2	26.7	42.5	**<0.001^*b*^**
FOX	12.2	24.0	36.0	**<0.001^*b*^**
CAZ	7.6	21.8	36.9	**<0.001^*b*^**
FTX	9.9	25.8	40.7	**<0.001^*b*^**
FEP	7.6	17.8	25.2	**<0.001^*b*^**
IMP	0.8	5.3	10.3	**<0.001^*b*^**
CIP	18.3	40.4	50.9	**<0.001^*b*^**
COL	0.0	0.4	2.8	**0.013^*b*^**
TGC	0.0	0.0	1.4	0.052
GEN	22.1	31.1	29.4	0.206
AMK	9.2	13.3	7.9	0.518
SXT	40.5	42.7	45.8	0.318

^*a*^ AUG, amoxicillin-clavulanate; ATM, aztreonam; CFZ, cefazolin; FOX, cefoxitin; CAZ, ceftazidime; FTX, cefotaxime; FEP, cefepime; IMP, imipenem; CIP, ciprofloxacin; COL, colistin; TGC, tigecycline; GEN, gentamicin; AMK, amikacin; SXT, trimethoprim-sulfamethoxazole.

^*b*^ *P* value <0.05 represents the statistically significant difference and is in bold.

In contrast, resistance to gentamicin (GEN), amikacin (AMK), trimethoprim-sulfamethoxazole (SXT), and tigecycline (TGC) remained relatively stable, without statistically significant changes across the study period. Although slight increases in resistance to colistin (COL) and TGC were noted (Fig. 2), these agents maintained relatively low resistance rates compared to other antibiotics. As gentamicin, amikacin, trimethoprim-sulfamethoxazole, and tigecycline did not show statistically significant changes across surveillance years, these antibiotics are not shown in Fig. 2.

### Antimicrobial susceptibility of the five most prevalent serotypes compared to other serotypes and non-typeable isolates

Based on serotyping, all isolates demonstrated intrinsic resistance to ampicillin (data not shown). The five most prevalent serotypes, K1, K2, K25, K62, and K64, exhibited a high variation in antibiotic resistance (Table 4). Serotypes K1 and K2 exhibited extremely low resistance to all tested antibiotics during 2006 and 2012. On the contrary, serotypes K64, K62, and K25 presented extremely high, high, and moderate resistance, respectively, to the tested antibiotics except for colistin and tigecycline (Table 4). Over 74% of serotype K64 isolates were resistant to all kinds of cephalosporins, with an overall of 25.6% resistant to carbapenem. In 2014–2018, resistance of serotype K64 isolates to quinolone increased to 100% resistance. Serotype K62 showed high resistance to third-generation cephalosporins, with resistance rates of 47.4% to ceftazidime and 50.0% to cefotaxime. Additionally, 10.5% of K62 isolates were resistant to carbapenems. Serotype K25 was the fifth most prevalent serotype in UTI, and moderate resistance was observed. Resistance to cephalosporins was between 17.4% and 26.1% but no resistance to carbapenem was identified. For other K-type serotypes, non-susceptibility rates to many antibiotics increased significantly from 2014 to 2018, indicating a broader rise in antibiotic resistance, as determined by the Cochran-Armitage trend test. Overall, resistance among UTI isolates increased during 2014–2018, and certain serotypes demonstrated fluctuating or consistently high resistance rates (Table 4).

**TABLE 4:** Antimicrobial susceptibility in urine culture isolates according to their serotypes

Serotype	Year	No. of isolates	Antibiotic non-susceptible rate (%)													
			AUG^*a*^	ATM	CFZ	FOX	CAZ	FTX	FEP	IMP	CIP	COL	TGC	GEN	AMK	SXT
K2	1998–2004	15	20.0	0.0	0.0	6.7	0.0	0.0	0.0	0.0	0.0	0.0	0.0	0.0	0.0	20.0
	2006–2012	17	5.9	0.0	5.9	5.9	5.9	5.9	0.0	5.9	5.9	0.0	0.0	0.0	0.0	0.0
	2014–2018	11	0.0	0.0	0.0	0.0	0.0	0.0	0.0	0.0	9.1	0.0	0.0	9.1	0.0	18.2
Total		43	9.3	0.0	2.3	4.7	2.3	2.3	0.0	2.3	4.7	0.0	0.0	2.3	0.0	11.6
K64	1998–2004	3	66.7	66.7	66.7	66.7	66.7	66.7	66.7	33.3	66.7	0.0	0.0	66.7	33.3	66.7
	2006–2012	18	88.9	83.3	88.9	83.3	83.3	83.3	77.8	27.8	94.4	0.0	0.0	83.3	66.7	88.9
	2014–2018	18	83.3	72.2	83.3	77.8	83.3	83.3	72.2	22.2	100.0	5.6	0.0	61.1	33.3	83.3
Total		39	84.6	76.9	84.6	79.5	82.1	82.1	74.4	25.6	94.9	2.6	0.0	71.8	48.7	84.6
K62	1998–2004	8	12.5	25.0	50.0	12.5	37.5	37.5	25.0	0.0	50.0	0.0	0.0	50.0	25.0	50.0
	2006–2012	19	84.2	47.4	63.2	52.6	57.9	63.2	42.1	15.8	73.7	5.3	0.0	68.4	42.1	68.4
	2014–2018	11	27.3	27.3	36.4	18.2	36.4	36.4	18.2	9.1	45.5	9.1	0.0	18.2	9.1	45.5
Total		38	52.6	36.8	52.6	34.2	47.4	50.0	31.6	10.5	60.5	5.3	0.0	50.0	28.9	57.9
K1	1998–2004	5	0.0	0.0	0.0	0.0	0.0	0.0	0.0	0.0	0.0	0.0	0.0	0.0	0.0	0.0
	2006–2012	9	22.2	0.0	11.1	11.1	0.0	11.1	0.0	0.0	0.0	0.0	0.0	0.0	0.0	0.0
	2014–2018	12	8.3	0.0	0.0	8.3	0.0	0.0	0.0	0.0	0.0	0.0	0.0	0.0	0.0	0.0
Total		26	11.5	0.0	3.8	7.7	0.0	3.8	0.0	0.0	0.0	0.0	0.0	0.0	0.0	0.0
K25	1998–2004	11	36.4	9.1	9.1	18.2	9.1	9.1	9.1	0.0	27.3	0.0	0.0	18.2	9.1	54.5
	2006–2012	8	62.5	25.0	37.5	37.5	25.0	37.5	25.0	0.0	37.5	0.0	0.0	37.5	0.0	75.0
	2014–2018	4	50.0	50.0	50.0	25.0	50.0	50.0	25.0	0.0	75.0	0.0	0.0	50.0	0.0	50.0
Total		23	47.8	21.7	26.1	26.1	21.7	26.1	17.4	0.0	39.1	0.0	0.0	30.4	4.3	60.9
Other K types^*b*^	1998–2004	72	18.1	11.1	19.4	11.1	5.6	5.6	5.6	0.0	16.7	0.0	0.0	22.2	9.7	23.6
	2006–2012	106	27.4	16.0	19.8	17.9	15.1	17.9	12.3	2.8	36.8	0.0	0.0	24.5	8.5	39.6
	2014–2018	113	35.4	24.8	36.3	31.0	27.4	32.7	19.5	12.4	43.4	0.9	0.0	23.9	2.7	37.2
Total		291	28.2	18.2	26.1	21.3	17.5	20.6	13.4	5.8	34.4	0.3	0.0	23.7	6.5	34.7
Non-typeable	1998–2004	17	11.8	5.9	17.6	0.0	0.0	5.9	0.0	0.0	11.8	0.0	0.0	23.5	5.9	23.5
	2006–2012	48	6.3	8.3	12.5	4.2	6.3	8.3	4.2	0.0	18.8	2.1	0.0	27.1	2.1	41.7
	2014–2018	45	44.4	31.1	48.9	35.6	46.7	35.6	26.7	0.0	48.9	4.4	48.9	33.3	4.4	53.3
Total		110	22.8	17.3	28.2	16.4	21.8	19.1	12.7	0.0	30.0	2.7	20.0	25.5	3.6	43.6

^*a*^ AUG, amoxicillin-clavulanate; ATM, aztreonam; CFZ, cefazolin; FOX, cefoxitin; CAZ, ceftazidime; FTX, cefotaxime; FEP, cefepime; IMP, imipenem; CIP, ciprofloxacin; COL, colistin; TGC, tigecycline; GEN, gentamicin; AMK, amikacin; SXT, trimethoprim-sulfamethoxazole.

^*b*^ K-antigen serotypes not included among those listed in the table.

### Distribution of virulence-associated determinants among different serotypes of isolates

The distribution of virulence genes among *K. pneumoniae* serotypes over the years is summarized in Table 5. Among the five most prevalent serotypes, K1 and K2 carried a significantly higher number of virulence genes compared to K25, K62, and K64. Specifically, for serotype K1, all tested virulence genes were present in ≥76.9% of the isolates except *clbA*. Although serotype K2 exhibited a slightly lower overall frequency of virulence genes compared to K1, ≥51.2% of K2 isolates still carried all tested virulence genes, excluding *clbA*. In contrast, serotypes K25, K62, and K64 demonstrated a relatively stable but consistently lower prevalence of virulence genes throughout the study period. Virulence gene expression in other K-type serotypes also remained low and stable. Notably, non-typeable isolates showed an almost complete absence of all tested virulence genes, suggesting they represent the least virulent group among *K. pneumoniae* isolates from urinary tract infections (Table 5).

**TABLE 5:** Virulence determinants among different serotypes of UTI *K. pneumoniae* isolates

Serotype	Year	No. of isolates	Virulent gene					
			*ClbA* (%)	*IroN* (%)	*RmpA* (%)	*RmpA2* (%)	*IutA* (%)	*IucA* (%)
K2	1998–2004	15	13.3	40.0	46.7	46.7	46.7	46.7
	2006–2012	17	23.5	70.6	70.6	64.7	70.6	70.6
	2014–2018	11	18.2	36.4	45.5	36.4	36.4	36.4
Total		43	18.6	51.2	55.8	51.2	53.5	53.5
K64	1998–2004	3	0.0	0.0	0.0	0.0	0.0	0.0
	2006–2012	18	0.0	11.1	5.6	5.6	5.6	5.6
	2014–2018	18	0.0	5.6	0.0	0.0	5.6	5.6
Total		39	0.0	7.7	2.6	2.6	5.1	5.1
K62	1998–2004	8	12.5	12.5	12.5	0.0	0.0	12.5
	2006–2012	19	21.1	26.3	26.3	26.3	21.1	5.3
	2014–2018	11	18.2	27.3	27.3	27.3	27.3	9.1
Total		38	18.4	23.7	23.7	21.1	18.4	7.9
K1	1998–2004	5	60.0	40.0	60.0	60.0	60.0	60.0
	2006–2012	9	66.7	77.8	77.8	77.8	77.8	77.8
	2014–2018	12	83.3	91.7	91.7	91.7	83.3	83.3
Total		26	73.1	76.9	80.8	80.8	76.9	76.9
K25	1998–2004	11	0.0	9.1	9.1	9.1	9.1	9.1
	2006–2012	8	0.0	0.0	0.0	0.0	12.5	12.5
	2014–2018	4	0.0	0.0	0.0	0.0	0.0	0.0
Total		23	0.0	4.3	4.3	4.3	8.7	8.7
Other K types^*a*^	1998–2004	72	5.6	13.9	16.7	19.4	15.3	15.3
	2006–2012	106	3.8	17.0	17.0	17.0	18.9	16.0
	2014–2018	113	5.3	18.6	17.7	17.7	21.2	21.2
Total		291	4.8	16.8	17.2	17.9	18.9	17.9
Non-typeable	1998–2004	17	0.0	0.0	0.0	0.0	0.0	0.0
	2006–2012	48	0.0	0.0	0.0	0.0	2.1	2.1
	2014–2018	45	0.0	0.0	0.0	0.0	0.0	0.0
Total		110	0.0	0.0	0.0	0.0	0.9	0.9

^*a*^ K-antigen serotypes not included among those listed in the table.

## DISCUSSION

UTIs are among the most common bacterial infections, with *K. pneumoniae* being the second leading causative pathogen after *Escherichia coli*. Although UTIs can generally be treated with antibiotics and have a low mortality rate, recurrent infections and reinfections are frequent, posing significant clinical and economic burdens (9). The overuse of antibiotics in managing recurrent UTIs contributes to the growing problem of antimicrobial resistance, making treatment increasingly challenging (10). Given these concerns, vaccination presents a promising long-term strategy to prevent UTIs by providing protective immunity against *K. pneumoniae* and reducing dependence on antibiotics. Developing effective vaccines could not only lower the incidence of recurrent infections but also mitigate the emergence of multidrug-resistant strains, ultimately improving patient outcomes and public health (11).

Previous efforts in *K. pneumoniae* vaccine development have primarily focused on CPS-based vaccines due to the capsule’s crucial role in bacterial virulence and immune evasion (12). CPS vaccines can be categorized into bioconjugate and non-bioconjugate types. Bioconjugate vaccines involve linking CPS to a carrier protein, which enhances immunogenicity and is particularly beneficial for long-term immunity (13). In contrast, non-bioconjugate CPS vaccines rely on the direct administration of purified polysaccharides, which can induce immune responses but may require adjuvants or booster doses for optimal effectiveness. Both CPS-based strategies show promise in providing broad protection against multiple *K. pneumoniae* serotypes, particularly those associated with invasive infections (14). However, effective vaccine development requires comprehensive serotype data to ensure adequate coverage. Recognizing this need, we previously conducted a survey on blood culture isolates. By integrating these data with urinary tract infection isolates, we can identify the most prevalent serotypes, allowing for the selection of optimal serotype candidates for vaccine formulation.

Our findings demonstrate important differences in serotype distribution between UTI and bloodstream infections, with implications for vaccine development. While some invasive serotypes (e.g., K1, K2, K20) were significantly more frequent in blood isolates, others, such as K64 and K28, were more associated with UTIs. Notably, non-typeable isolates were more common in UTIs than in blood cultures, although the difference was not statistically significant (19.3% vs 14.8%, *P* = 0.057). These results highlight the potential need for infection-site-specific vaccine strategies and support the prioritization of certain serotypes, such as K64, for inclusion in future CPS-based vaccines. Integration of data from multiple infection sites, as done here, enhances our ability to identify the most prevalent and clinically relevant serotypes for effective vaccine design.

In addition, when compared with the findings of Kao et al. (15), our data revealed both similarities and differences in serotype distribution patterns. While their single-center study reported a dominance shift from K1 to K64 over 24 years, our multicenter data confirmed the continued prevalence of K64, K2, and K1, and additionally identified K62 and K25 as prominent serotypes not emphasized in their findings (15). This likely reflects differences in geographic coverage and patient populations. Our broader sampling from multiple centers minimizes institutional bias and better represents the diversity of community-onset UTIs. Furthermore, several serotypes were consistently absent across two decades of surveillance, underscoring their limited role in these infections. These observations further highlight the importance of region-specific, large-scale surveillance in informing vaccine design and public health strategies.

For the antimicrobial susceptibility test on those *K. pneumoniae* isolates with UTI, significant changes in antibiotic resistance from 1998 to 2018 were observed. Significant increases in resistance were observed for several antibiotics, including ATM, different generations of cephalosporins, including CFZ, FOX, CAZ, FTX, FEP, and IMP, CIP, AUG. The resistance rates for these antibiotics significantly increased (Cochran-Armitage trend test, *P* < 0.001) over the years, indicating a growing challenge in the regimen of *K. pneumoniae* UTI infections. On the other hand, resistance levels for GEN, AMK, SXT, and TGC showed no significant change. However, COL demonstrated a significant increase in resistance during the surveillance period. These findings highlight the escalating challenge of antimicrobial resistance in *K. pneumoniae* UTI infection, emphasizing the urgent need for infection control by vaccinating high-risk groups and novel therapeutic strategies.

Our investigation into *K. pneumoniae* serotypes and their antimicrobial susceptibility patterns provides important insights aligned with global concerns regarding emerging resistance trends. These concerns are well captured in Table 2, “Predicted Issues in Gram-Negative Bacteria Resistance in the Next Decade,” from the article “How Soon Is Now? The Urgent Need for Randomized, Controlled Trials Evaluating Treatment of Multidrug-Resistant Bacterial Infection” by Paterson and Roger (16). Their projections regarding the increase in carbapenem-resistant organisms and the heightened reliance on last-line antibiotics, such as polymyxins and tigecycline, are reflected in our findings, particularly the rise in antimicrobial resistance among K-type serotypes between 2014 and 2018.

In our study, serotypes K62 and K64 demonstrated notably high resistance rates, with K64 exhibiting over 74.4% resistance across all tested cephalosporins. These findings are consistent with global observations of increasing resistance among Gram-negative pathogens, especially in community-acquired infections, where treatment options using oral agents are becoming increasingly limited (17, 18).

A noteworthy finding is the serotype-dependent variation in antimicrobial susceptibility among *K. pneumoniae* isolates, highlighting the need for a nuanced approach in developing CPS-based vaccines. Among the five most prevalent serotypes, K1 and K2 generally exhibited higher susceptibility to antibiotics compared to other serotypes and non-typeable isolates, indicating that infections caused by these vaccine-targeted strains may remain more responsive to conventional antimicrobial therapy. However, while antibiotics may effectively treat initial infections, they do not offer long-term protection for patients with recurrent or relapsing UTIs. Notably, serotypes K62 and K64 demonstrated higher resistance to first-line antibiotics, with 52.6% and 84.6% resistance to cefazolin, respectively. K64, in particular, exhibited over 74.4% resistance across all tested cephalosporins. The marked rise in antimicrobial resistance among various K-type serotypes between 2014 and 2018 further emphasizes the importance of broad serotype coverage in vaccine design. When analyzing the correlation between serotype and virulence genes, findings were consistent with previous studies (15) and blood culture analyses (8). K1 and K2 carried the highest number of virulence genes, whereas other serotypes harbored fewer. Our findings further demonstrated that non-typeable isolates exhibited minimal or no virulence genes, suggesting their low pathogenic potential, which supports the role of the capsule as a major virulence determinant (19).

This study has several limitations. First, due to the nature of the Taiwan Surveillance of Antimicrobial Resistance (TSAR) system, clinical metadata, such as patient age, sex, comorbidities, and prior antibiotic exposure, were not available. Therefore, we were unable to evaluate whether host-related factors contributed to the observed differences in serotype distribution and antimicrobial resistance between urine and blood isolates. Second, although the blood culture isolates used for comparison were derived from the same TSAR program, detailed geographic and demographic matching between specimen sources was not feasible. These limitations may affect the interpretation of trends and restrict the ability to generalize findings to specific patient populations. Future studies incorporating patient-level clinical data are warranted to better clarify the relationship between host characteristics, infection site, and serotype distribution.

A previous study reported the development and phase I clinical evaluation of a 24-valent *K. pneumoniae* CPS vaccine, demonstrating both the feasibility and safety of producing a multivalent CPS-based vaccine (7). The selection of CPS types for vaccine formulation should be guided by the prevalence of antimicrobial-resistant clones at both local and global levels. By integrating data from previous blood culture surveillance with findings from the present study, we observed that the distribution of *K. pneumoniae* serotypes causing infections has remained relatively stable over time. Therefore, CPS type selection can be based on primary surveillance data, with periodic monitoring to detect any shifts in serotype prevalence, similar to the strategy used for *Streptococcus pneumoniae* vaccines. In conclusion, this study provides valuable insights into the use of CPS serotype distribution as a strategic approach for controlling *K. pneumoniae* infections through vaccination.

## MATERIALS AND METHODS

### Collection of isolates

TSAR is a laboratory-based passive surveillance program coordinated by the National Health Research Institutes since 1998. Every other year, participating hospitals across Taiwan are invited to submit non-duplicate, clinically significant bacterial isolates from various clinical specimens. In this study, we analyzed *K. pneumoniae* isolates recovered from outpatient urine cultures submitted between 1998 and 2018. These isolates were considered representative of community-onset UTIs. A total of 570 non-repetitive *K. pneumoniae* urine isolates were included, with 131, 225, and 214 isolates collected from the periods 1998–2004, 2006–2012, and 2014–2018, respectively. All isolates were obtained through the TSAR program following consistent protocols across the surveillance years.

For comparative analysis, we included data from a previously reported cohort of 521 *K*. *pneumoniae* isolates obtained from blood cultures through the same TSAR program. These bloodstream isolates were collected in three corresponding surveillance years: 1998 (*n* = 121), 2008 (*n* = 197), and 2018 (*n* = 203) (8). All isolates were non-duplicate and submitted by the same set of hospitals participating in the TSAR program, allowing consistency in geographic and methodological context.

### Serotyping by rapid antigen tests, PCR typing, and sequencing

The isolates were serotyped by rapid cassette to classify the K1, K2, and non-K1/K2 groups (20). We used serotype-specific primer sets to perform multiplex PCR for serotyping. If non-typable isolates were observed from the first two serotyping tests, then *wzi, wza,* and *wzc* gene sequencing was performed to confirm the serotype. Isolates that tested negative for both cassette typing and wzi/wza/wzc sequencing were classified as non-typeable. PCR and sequencing of these genes were performed according to Table S1 (21–24).

### Virulence-associated gene detection and antibiotic susceptibility testing

The genes *iroN*, *clbA*, *entB*, *rmpA*, *rmpA2*, *iutA,* and *iucA* were detected to study the virulence of *K. pneumoniae*. The primer set is listed in Table S2 (25, 26). The Taq for *entB* was *amaR* One PCR HotStar, Taiwan, and the other virulence factors, *iroN*, *clbA*, *rmpA*, *rmpA2*, *iutA,* and *iucA*, used Q-Amp 2X ScreeningFire Taq Master Mix, Taiwan. The reactions were subjected to 30 amplification cycles, with annealing temperatures ranging from 55°C to 57°C, optimized for each primer pair.

### Antimicrobial susceptibility testing

We tested the susceptibility of isolates to amikacin, gentamicin, amoxicillin-clavulanate, piperacillin-tazobactam, ampicillin, cefazolin, ceftazidime, cefepime, cefoxitin, cefuroxime, cefotaxime, imipenem, meropenem, ciprofloxacin, trimethoprim/sulfamethoxazole, and aztreonam by means of the broth microdilution method. Susceptibility of isolates to tigecycline and colistin was determined by E-test. The minimal inhibitory concentration results were interpreted following the standard of the Clinical and Laboratory Standards Institute (27), except for tigecycline and colistin. The Food and Drug Administration breakpoint was used for tigecycline (28), and for colistin, we used the European Committee on Antimicrobial Susceptibility Testing breakpoint (29).

### Statistical analysis

The chi-square test was used to compare the proportions of isolates from blood and urine among different bacterial serotypes. A significant chi-square result indicates that the distribution of serotypes differed between blood and urine sources. The Cochran-Armitage trend test was used to evaluate temporal trends in antimicrobial susceptibility across surveillance years. A significant trend result indicates that the susceptibility of a given serotype to an antibiotic increased or decreased over time. Statistical significance was defined as *P* < 0.05. All analyses were performed using R software (version 4.3).

## SUPPLEMENTAL MATERIAL

Tables S1 and S2 spectrum.02633-25-s0001.docx Table S1: Primer sets used in this study for the identification of *K. pneumoniae* serotypes. Table S2: Virulence-associated genes detected in this study.

ASM does not own the copyrights to Supplemental Material that may be linked to, or accessed through, an article. The authors have granted ASM a non-exclusive, world-wide license to publish the Supplemental Material files. Please contact the corresponding author directly for reuse.
