# Supplementary material for: The sero-prevalence and virulence determinants of Klebsiella pneumoniae isolated from urine in outpatient units: a 20-year collection from Taiwan as a herald for serotype vaccine selection
Source: Microbiol Spectr. 2025 Dec 11;14(2):e02633-25. doi: 10.1128/spectrum.02633-25 (PMC12889052; doi:10.1128/spectrum.02633-25)
Supplement: Tables S1 and S2 — Table S1: Primer sets used in this study for the identification of K. pneumoniae serotypes. Table S2: Virulence-associated genes detected in this study. [file spectrum.02633-25-s0001.docx]

Supplementary Table 1. Primer sets used in this study for the identification of *K. pneumoniae* serotypes

| Serotype | Primer name | Sequence | Expected amplicon | Accession no. | Reference |
| --- | --- | --- | --- | --- | --- |
| K1 | wzyK1F  wzyK1R | 5′-GGTGCTCTTTACATCATTGC-3′  5′-GCAATGGCCATTTGCGTTAG-3′ | 1283 bp | AB924547 | (23) |
| K2 | wzyK2F  wzyK2R | 5'-GACCCGATATTCATACTTGACAGAG-3'  5'-CCTGAAGTAAAATCGTAAATAGATGGC-3' | 641 bp | AB371296 | (23) |
| K3 | WzyK3F  WzyK3R | 5'-TAGGCAATTGACTTTAGGTG-3'  5'-AGTGAATCAGCCTTCACCT-3' | 549 bp | FQ311478 | (30) |
| K4 | K4wzcF  K4wzcR | 5'-CATCATCGGGATTACAGC-3'  5'-GGCAGGCACATTGAGACG-3' | 337 bp | AB924548 | This study |
| K5 | K5wzxF  K5wzxR | 5'-TGGTAGTGATGCTCGCGA-3'  5'-CCTGAACCCACCCCAATC-3' | 280 bp | AB371292 | (23) |
| K5 | K5wzcF  K5wzcR | 5'-CTTAGTTCTCGTTCGTG-3'  5'-AGCCCATTATCATTACTC-3' | 426 bp | AB371292 | This study |
| K6 | K6wzcF  K6wzcR | 5'-ATGACTGAAAGAACCAAGCA-3'  5'-TGATCGTATTCTAGCCGTG-3' | 350 bp | AB924549 | This study |
| K7 | K7wzyF  K7wzyR | 5'-TCGCAAGAGTTAGTTATT-3'  5'-CCTATCAGAGGCAAAAG-3' | 960 bp | AB924550 | This study |
| K8 | K8wzyF  K8wzyR | 5'-AGGGGATAGCGTAGGTA-3'  5'-TAGAATGCCCAAACTGT-3' | 933 bp | AB924551 | This study |
| K9 | K9wzyF  K9wzyR | 5'-ATGGTGATTATGAATGAAG-3'  5'-ACACAATGAAAACATTGCC-3' | 1283 bp | AB371293 | (22) |
| K10 | K10wzyF  K10wzyR | 5'-GTATTATTCAGTATGTCGCT-3'  5'-GCAAAAGTAGATGAGGTTA-3' | 644 bp | AB924552 | This study |
| K11 | K11wzcF  K11wzcR | 5'-TTGATGAAGCCGTTACAC-3'  5'-ACCAGCATTCGGACTAG-3' | 393 bp | AB924553 | This study |
| K12 | K12wzyF  K12wzyR | 5'-TCGTCTTTACTACCTCCG-3'  5'-AATACCCCTGACACTGC-3' | 576 bp | AB924554 | This study |
| K13 | K13wzyF  K13wzyR | 5'-TTATTTGCTTACCTTGC-3'  5'-TATGTCTGAACCTCACTAA-3' | 768 bp | AB924555 | This study |
| K14 | K14wzyF  K14wzyR | 5'-GACTCTGAATAAAAGAACAC-3'  5'-CTCAATAAATCTGTTCTGAAG-3' | 1209 bp | AB371294 | (22) |
| K15 | K15wzyF  K15wzyR | 5'-TACCCATAGCTATATGCGGC-3'  5'-GGGAAAGTTGCAGCATATTC-3' | 800 bp | AB924556 | (22) |
| K16 | K16wzyF  K16wzyR | 5'-ATGGTACCGTTGGGGTTATC-3'  5'- TAATCAACAATGTCGTAGCG-3' | 742 bp | AB742228 | (22) |
| K17 | K17wzyF  K17wzyR | 5'-TAATGGGGACGAATCAG-3'  5'-CGCTAACGAATAGACCA-3' | 500 bp | AB924557 | This study |
| K18 | K18wzyF  K18wzyR | 5'-GAGGATGTGACAAAGCC-3'  5'-AACCCTAACCCTAAACC-3' | 611 bp | AB924558 | This study |
| K19 | K19wzyF  K19wzyR | 5'-GTTATGTAATAGGCTTTTC-3'  5'-TATTCGCACCATAGTTC-3' | 654 bp | AB924559 | This study |
| K20 | K20wzyF  K20wzyR | 5'-CGGTGCTACAGTGCATCATT-3'  5'-GTTATACGATGCTCAGTCGC-3' | 741 bp | AB371289 | (30) |
| K21 | K21wzyF  K21wzyR | 5'-TTCTTAGTTTGTTGGTGT-3'  5'-TTACTTGCTCTGCTGTT-3' | 577 bp | AB924560 | This study |
| K22 | K22wzcF  K22wzcR | 5'-ATTCTGGGATGCTTACTC-3'  5'-ACCTATCGCACTTGACTT-3' | 539 bp | AB819893 | This study |
| K23 | K23wzyF  K23wzyR | 5'-TGAACTATTCGGTTTACG-3'  5'-CTATTTGCTCCAACACTA-3' | 951 bp | AB924561 | This study |
| K24 | K24wzcF  K24wzcR | 5'-TGTTGAGCGACAAGCAGC-3'  5'-TCAGCAGGATTAGGAGGC-3' | 1074 bp | AB924562 | This study |
| K25 | K25wzyF  K25wzyR | 5'-GTTTACTCTGCTCTATTTAC-3'  5'-TTCAACCTTCTCCTACT-3' | 902 bp | AB924563 | This study |
| K26 | K26wzyF  K26wzyR | 5'-TAGTCTGGGTCTATCTGA-3'  5'-ACTGTGACAACAAATGG-3' | 1101 bp | AB924564 | This study |
| K27 | K27wzcF  K27wzcR | 5'-TCAGCCTCAATCAGCACC-3'  5'-TTCAAACCTGGCAACAAG-3' | 1250 bp | AB924565 | This study |
| K28 | K28wzyF  K28wzyR | 5'-GCGTGACACTTCGTTAT-3'  5'-TCCACCGATACCACCAT-3' | 683 bp | AB924566 | This study |
| K29 | K29wzxF  K29wzxR | 5'-AGAACAGGCACTATTACG-3'  5'-GCTTACAAGATACCGAAA-3' | 763 bp | AB924567 | This study |
| K30 | K30wzyF  K30wzyR | 5'-CAACTAATGTTTCCCTCG-3'  5'-TAGATTGCATAACCCTCA-3' | 738 bp | AB924568 | This study |
| K31 | K31wzyF  K31wzyR | 5'-TAACAATCACTTTATCGCTG-3'  5'-CAAAGCTGACATACAAATGA-3' | 442 bp | AB924569 | This study |
| K32 | K32wzyF  K32wzyR | 5'-ATGAGGGTTATTAGCAATCT-3'  5'-CAGATTCACCCGTTTAAATA-3' | 564 bp | AB924570 | This study |
| K33 | K33wzyF  K33wzyR | 5'-TATTGCCCATAACGAGC-3'  5'-GATAACCGATGAGCGAC-3' | 1090 bp | AB924571 | This study |
| K34 | K34wzyF  K34wzyR | 5'-AGATGGCTTTACAATGC-3'  5'-CAGGATGGAAAACAGTG-3' | 582 bp | AB924572 | This study |
| K35 | K35wzcF  K35wzcR | 5'-CCTGGTGCTGTATTTGAT-3'  5'-CCTGATTCTACATCCCTAC-3' | 560 bp | AB924573 | This study |
| K36 | K36wzyF  K36wzyR | 5'-GCATAATGACACAGAGAGGA-3'  5'-AATGGATTAGTGCCGCTATC-3' | 351 bp | AB924574 | This study |
| K37 | K37wzyF  K37wzyR | 5'-CCTTAGCAATACCAACT-3'  5'-CAATAACAAACCTCCAC-3' | 804 bp | AB924575 | This study |
| K38 | K38wzyF  K38wzyR | 5'-GATGAGGGCATAAGTAA-3'  5'-AATGAAATAGCGATAAGA-3' | 527 bp | AB924576 | This study |
| K39 | K39wzyF K39wzyR | 5'-ATGACCAATGACTTACAAAG-3'  5'-GAATTCCGTTCCAGCCCAC-3' | 1100 bp | AB742230 | (22) |
| K40 | K40wzyF  K40wzyR | 5'-GAGAACAACTACGGCTTT-3'  5'-CACCACAATAAAACTAACC-3' | 503 bp | AB924577 | This study |
| K41 | K41wzyF  K41wzyR | 5'-TCTTTGCTACATCAGGA-3'  5'-TAATAACCCACAGGACA-3' | 879 bp | AB924578 | This study |
| K42 | K42wzcF  K42wzcR | 5'-TACCCGATAGCCAACCAC-3'  5'-AGACAGCACGCCCAGATT-3' | 924 bp | AB924579 | This study |
| K43 | K43wzyF  K43wzyR | 5'-CGACTTTGGTTTCCTTC-3'  5'-CTTGACCTTTCCTTTCC-3' | 532 bp | AB924580 | This study |
| K44 | K44wzcF  K44wzcR | 5'-CAGACAGCCAACCACAAT-3'  5'-AATACACGCAGAAGCACC-3' | 1108 bp | AB924581 | This study |
| K45 | K45wzyF  K45wzyR | 5'-TGTATGCTCTTAGTCTTTC-3'  5'-CTCAGTAATCCTTTTCG-3' | 842 bp | AB924582 | This study |
| K46 | K46wzyF  K46wzyR | 5'-GACAAAACCTGTATCACTG-3'  5'-AAAACCTATTAGCACGC-3' | 988 bp | AB924583 | This study |
| K47 | K47wzyF2  K47wzyR2 | 5'-TACACAAGGGAAAATTAACATCACC-3'  5'-CCCTGGGATAGGAAAATTTCAATAT-3' | 961 bp | AB924584 | This study |
| K48 | K48wzcF  K48wzcR | 5'-GGGACGAGCGGTTTACAT-3'  5'-TATTATTGCGGCATCAGT-3' | 823 bp | LT174559 | This study |
| K49 | K49wzyF  K49wzyR | 5'-GTCGAGCTACTACCGTTAGG-3'  5'-CACGTATAGCTTTCATCAGC-3' | 733 bp | AB924586 | This study |
| K50 | K50glyF  K50glyR | 5'-CCAATGATAATACTGCGCAG-3'  5'-CAACCCGATCATATCATCTC-3' | 616 bp | AB924587 | (22) |
| K51 | K51wzyF  K51wzyR | 5'-TATGGGAGTTGAAGTAGA-3'  5'-GTAAAGCGGAGATAAGTG-3' | 379 bp | AB924588 | This study |
| K52 | K52F  K52R | 5'-GACAAAGTCACCAGCAA-3'  5'-CCAAATCGCAGTAACAC-3' | 500 bp | AB924589 | This study |
| K53 | K53wzyF  K53wzyR | 5'-TGTTCGTTTCATTTGAC-3'  5'-AATACCAGGAGAAGATAGTA-3' | 395 bp | AB924590 | This study |
| K54 | K54wzyF  K54wzyR | 5'-TTACCTCAGAGCGTTGCATTG-3'  5'-TTAGGTATGACAATTGAGCTC-3' | 953 bp | AB924591 | (22) |
| K55 | K55wzcF  K55wzcR | 5'-TATTGATGCCGATTTACG-3'  5'-ATTGGAACGCCACTTTGT-3' | 390 bp | AB924592 | This study |
| K56 | K56wzyF  K56wzyR | 5'-TTCGGAATGGTTGAGTA-3'  5'-AACAAGTCGTTTAGATAGG-3' | 891 bp | AB924593 | This study |
| K57 | K57wzyF  K57wzyR | 5'-CTCAGGGCTAGAAGTGTCAT-3'  5'-CACTAACCCAGAAAGTCGAG-3' | 1037 bp | AB924594 | (28) |
| K58 | K58wzcF  K58wzcR | 5'-GACAAATAAAGGCGGTAA-3'  5'-CTTCAAATAGAGCGACCA-3' | 560 bp | LT174567 | This study |
| K59 | K59wzcF  K59wzcR | 5'-CTAAAGGGAAACGGAAGA-3'  5'-CAAGTATTGGCGGTGTAT-3' | 498 bp | AB924596 | This study |
| K60 | K60wzyF  K60wzyR | 5'-TGTACTAGTTAGTGTGCCCG-3'  5'-GCATCTACGGAATATCTTGA-3' | 500 bp | AB924597 | This study |
| K61 | K61wzyF  K61wzyR | 5'-GCTAATGCTGCTGGTTGT-3'  5'-GTTATGCGGTGGGTAAAT-3' | 447 bp | AB924598 | This study |
| K62 | K62wzyF  K62wzyR | 5'-ATGTCAGTGATTATTTCAGG-3'  5'-AGAGTATGTCATCACGCACG-3' | 1050 bp | AB371295 | (22) |
| K63 | K63wzcF  K63wzcR | 5'-TGTTTGGAAAGGGATGGG-3'  5'-ATGAAGGCTGGTGCGTAA-3' | 1202 bp | AB924599 | This study |
| K64 | K64wzyFnew  K64wzyRnew | 5'-CTTTTAGGGCTACGGCACC-3'  5'-CCGCGCGCAGGAACATTAG-3' | 700 bp | AB924600 | (31) |
| K65 | K65wzyF  K65wzyR | 5'-GGATTACTAACTCAAACAC-3'  5'-ATAAGAAAGAAACGACTAG-3' | 521 bp | AB924601 | This study |
| K66 | K66wzyF  K66wzyR | 5'-TTGTTTTGCGATTCTTA-3'  5'-GAGTCATTAGCCCAGTC-3' | 563 bp | AB924602 | This study |
| K67 | K67wzyF  K67wzyR | 5'-ATCTTCAGAACATGATCCGG-3'  5'-TCAGCAATACCAAAGCAATT-3' | 902 bp | AB924603 | This study |
| K68 | K68wzyF  K68wzyR | 5'-GAAGGGTGTTCGTAGTA-3'  5'-CAACTCAGGAAGAATAAC-3' | 835 bp | AB924604 | This study |
| K69 | K69wzcF  K69wzcR | 5'-TGAACAATGAAGAGGGAA-3'  5'-ACCAAGCCAATAAGAACA-3' | 868 bp | AB720689 | This study |
| K70 | K70wzyF  K70wzyR | 5'-TAATATTTATTGCTGCGTGC-3'  5'-CAGCATTGCAATAAGTATGA-3' | 401 bp | AB924606 | This study |
| K71 | K71wzxF  K71wzxR | 5'-TTTCTCGGCAGGTGTCT-3'  5'-TCGGTCAAAAGTGTCGC-3' | 540 bp | AB924607 | This study |
| K72 | K72wzcF  K72wzcR | 5'-TTTAGGTCGTCTTATTGG-3'  5'-ACTTCGCACTTCAGGTAG-3' | 787 bp | AB924608 | This study |
| K74 | K74wzyF  K74wzyR | 5'-AACACTAGAAACTATACATCCGT-3'  5'-AAAGCCTCCCAATCCAG-3' | 996 bp | AB924609 | This study |
| K79 | K79wzcF  K79wzcR | 5'-CAGGAGATAAAGCGGGTA-3'  5'-CTGGTTTGGGTTGGGTCA-3' | 873 bp | AB924610 | This study |
| K80 | K80wzyF  K80wzyR | 5'-ATTTTAGCATTTACCATCC-3'  5'-ATCCCATCCATTCACAA-3' | 813 bp | AB924611 | This study |
| K81 | K81wzyF  K81wzyR | 5'-TCTTATGCGGTTGTCTGT-3'  5'-CTGATGTAAGCGTGAATG-3' | 374 bp | AB924612 | This study |
| K82 | K82wzyF  K82wzyR | 5'-ATGTTACCCTATGTCTTAGTTC-3'  5'-ACACCACTAAAGAAGAATGT-3' | 404 bp | AB924613 | This study |
| KN1 | KN1F  KN1R | 5'-ATTGGGATAATGATGGTTTG-3'  5'-ATAGGAACAGCCCAGTAAAA-3' | 890 bp | AB924614 | This study |
| KN2 | KN2F  KN2R | 5'-TGTCCTTAGTCACAAATAAGTC-3'  5'-CTCTTTGGTATATAGCTGTGAA-3' | 1004 bp | AB371290 | This study |
| *Wzi* | wzi | 5′-GTGCCGCGAGCGCTTTCTATCTTGGTATTCC-3′  5′-GAGAGCCACTGGTTCCAGAA[C or T]TT[C or G]ACCGC-3′ | 580 bp |  | (21) |
| *Wza* |  | 5′-TGAAAGTGTTTGTCATGGG-3′  5′-TTCAGCTGGATTTGGTGG-3′  5′-GCTTCCATCATTGCAAAATG-3′ |  |  | (22) |
| *Wzc* |  | 5′-GGGTTTTTATCGGGTTGTAC-3′  5′-TTCAGCTGGATTTGGTGG-3′  5′-GCTTCCATCATTGCAAAATG-3' |  |  | (22) |

Supplementary Table 2. Virulence-associated genes detected in this study and performed as previously published (25)

| Target gene | Primer name | Primer used | Expected amplicon (bp) |
| --- | --- | --- | --- |
| *clbA* | ClbA 1F | ATGAGGATTGATATATTAATTGGACA | 735 |
|  | ClbA 1R | TCAATTCTGCCCATTTGACG |  |
| *entB* | entB-F | ATTTCCTCAACTTCTGGGGC | 371 |
|  | entB-R | AGCATCGGTGGCGGTGGTCA |  |
| *iroN* | iroN-F | GTCCGGCGGTAACTTCAGCC | 829 |
|  | iroN-R | TCAGAATGAAACTACCGCCC |  |
| *iucA* | iucA-F | ATAAGGCAGGCAATCCAG | 2927 |
|  | iucA-R | TAACGGCGATAAACCTCG |  |
| *iutA* | iutA-F | GGCTGGACATCATGGGAACTGG | 300 |
|  | iutA-R | CGTCGGGAACGGGTAGAATCG |  |
| *rmpA* | rmpA-F | TACATATGAAGGAGTAGTTAAT | 505 |
|  | rmpA-R | GAGCCATCTTTCATCAAC |  |
| *rmpA2* | rmpA2-F | TGTGCAATAAGGATGTTACATTAGT | 609 |
| *rmpA2* | rmpA2-R | TTTGATGTGCACCATTTTTCA |  |

**References**
30. Zhang S, Yang G, Ye Q, Wu Q, Zhang J, Huang Y. 2018. Phenotypic and genotypic characteGenotypic Characterization of Klebsiella pneumoniae isolated from retail fooIsolated From Retail Foods in China. Front Microbiol 9:289. <https://doi.org/10.3389/fmicb.2018.00289>

31. Pan YJ, Lin TL, Lin YT, Su PA, Chen CT, Hsieh PF, Hsu CR, Chen CC, Hsieh YC, Wang JT. 2015. Identification of capsular types in carbapenem-resistant Klebsiella pneumoniae strains by wzc sequencing and implications for capsule depolymerase treatment. Antimicrob Agents Chemother 59:1038–1047. <https://doi.org/10.1128/AAC.03560-14>
